# Obsolescence and Intervention: On Synthetic-Biological Entities

**DOI:** 10.3389/fbioe.2014.00059

**Published:** 2014-11-26

**Authors:** Andrés Moya

**Affiliations:** ^1^Cavanilles Institute of Biodiversity and Evolutionary Biology, University of Valencia, Valencia, Spain; ^2^Valencian Region Foundation for the Promotion of Health and Biomedical Research (FISABIO), Valencia, Spain; ^3^Consortium for Biomedical Research in Epidemiology and Public Health (CIBEResp), Madrid, Spain

**Keywords:** natural entities, artificial entities, metabolism, evolution, machines

Oftentimes, topics that might fall outside of science’s remit seem to end up becoming a part of it, sooner or later. This appears to be the case of synthetic biology, a new biological science (although some maintain that it is a form of engineering, or treat it as such; Endy, 2005), which seems to have become essential to the understanding of living beings and their extreme manipulation. I believe it to be a new form of biology. In truth, synthetic biology has a long history and, conceptually speaking, may well have formed part of the interests and research efforts of our illustrious predecessors throughout the first half of the twentieth century and even earlier. In any event, and broadly speaking, it may be asserted that such efforts were premature and that the state of the science, at that time, did not allow for progress, in terms of the modification, creation, or re-creation of organisms or parts thereof that we have today. Now, it has rather come of age, which is why I think of synthetic biology as a young or new biological science (Moya, 2014).

To a great extent, when you talk about synthetic biology, what you are doing, first and foremost, is making a statement of intent, if not expressing your concern, wish, or hope that any biological organism which this discipline might examine should be completely under control, and that it should not deviate even the slightest bit from the role that has been ascribed to it. Of course, if some achievements had not already been made in the field of synthetic biology, clinging to a mere statement of intent would do little to further its chances of survival in the future. If some sciences make headway, it is because some of their initial achievements thrust fame, awareness, and recognition upon them, in the eyes of the scientific community and the rest of society. Here is where we start to get to grips with wishes and hopes for these new sciences and their potential. This is what is happening with synthetic biology.

Fundamentally, the synthetic biologist aspires to make manufactured biological entities behave just as a car might, once it has been put together on an assembly line. The metaphor of the car is as valid as that of any other mechanical entity, or any other type of entity, with perhaps the only condition being that they should all be put together on an assembly line. A car, effectively, begins to take shape on an assembly line and ends up having a specific physical form. It comprises many different components and each one has a specific function, as designated by the manufacturer. Together, its components combine to form a mechanical entity that functions in a pre-determined way because of prior knowledge of the manner in which each component works, and the manufacturer puts them all together following a pre-determined plan, so that the whole may function as desired. Attempting to make a manufactured biological entity function in the same way as a car leads us to make two relevant observations. To begin with, there is the obsolescence of the entity, then there is intervention in the entity itself. The nature of these properties differs in cells when they are compared to cars.

First, let us take a look at the question of obsolescence. As everyone knows, a car has a limited lifespan. Its constituent parts, in particular those that are essential in order for it to run, become worn down, inevitably, until the materials [from which they are made] break down and become altered through the contact of some parts with others, or on account of the various reasons for which the car might cease to run. I refer not to the failure of the car to run, on account of damage incurred following an accident, but quite simply to wear and tear, rendering it unable to perform the purpose for which it has been intended. To what degree might this metaphor apply to the biological entity, in general, and the synthetic-biological entity, in particular? In truth, the following question might be asked, equally, of the biological entity and the car. Might it suffer some type of wear and tear that causes it either to stop functioning or to function in a different way before, for example, reaching the culminating point of its division or reproduction? In other words, before it can reproduce itself? This is, in effect, the case. Let us take, for example, an individual biological entity; a microorganism or a cell from a multicellular organism which, on account of its specialization, could be a germ cell – the purpose of which is the reproduction of the organism – or a non-germ cell, which will subdivide through mitosis, creating copies of itself. In the case of the microorganism, or the non-germ cells, there comes a point at which they divide, producing two genetic copies of themselves; however, up until that point, they have undergone transformation processes, ending with metabolism, that have altered them in relation to what they might have become in the moment that they were generated by their respective parent cells. It is generally believed that it is only at the point of division (reproduction), when genetic changes occur, that they are at their most relevant, when considering any possible transformation. These changes manifest themselves once they start to exhibit genetic differences from their progenitors; however, what I am talking about here are previous changes in the metabolic machinery. The cell is transformed, grows, and, before multiplying, changes. Consequently, this is not just about genetic changes; it is also about changes in the cell’s metabolism (de Lorenzo, 2014). Contrary to cars, that cannot avoid wear and tear, cells are undergoing metabolic changes to avoid obsolescence up to a point. A microbial cell is usually said to be immortal, in that before it ceases to exist in that form, it has already divided. And in a multicellular organism, with specialized reproductive cells, it is usually these cells themselves that join with the reproductive cells of other organisms, having previously undergone meiosis, in order to reproduce. Yet, all of these different types of cells – the immortal microbial cells, the germ cells that have the capacity to reproduce, and all the other non-germ cells that undergo mitosis – transform metabolically, before reaching their respective points of division and reproduction. And these changes, which are quite noticeable and dramatic, can be crucial. So much so that they may end the very life of the corresponding entities before division or reproduction can take place. All of these cells have a lifespan that has been optimized, through evolution, to ensure that they reach the point of division or reproduction, whichever is relevant. We can, it seems, only take the metaphor of the cell as car so far. There is something that does not quite fit, because eventually cars become obsolete and end up in the scrapyard, whereas cells, even though they have a limited lifespan, and undergo transformation and decomposition eventually, are able to reproduce. They arrive, it would seem, at these stages, with a certain degree of autonomy. The higher autonomy displayed by living systems has its basis in their internal organizational dynamics, that is, in the fact that cells (and organisms in general) are self-maintaining, self-organizing, self-repairing, and self-reproducing systems (Nicholson, 2013). It is these properties that confer organisms a far greater degree of functional autonomy when compared to machines. The metaphor of the car would only be valid if it were possible to extend the lifespan of its component parts and if, by means of systematic repair, the car continued to be the same car that rolled off the assembly line. In order for the car to remain the same, intervention must take place. And here is where we come to our second observation, the biological entity – in order to remain the same and reach the point of division or reproduction – effects its own intervention, regulating itself by means of its metabolism. All of evolution – more than 3000 million years of ceaseless effort – has conspired to ensure that this self-driven intervention of single-cell organisms, which divide, and multicellular organisms, which have cells that divide and others that reproduce, is effective; in other words, ensuring that the transformations that take place as a result of these organisms being in permanent interaction with their environment do not do so to such an extent that they cause them to decompose or degenerate before they can divide or reproduce (Danchin, 2009). The obsolescence of a cell is remedied by self-intervention, whereas the wear and tear of a car is externally remedied. The crucial difference with regards to intervention seems to be that cars require external intervention to remain viable and operational whereas cells have internal means of repairing damaged parts and compensating against external perturbations. This observation, which I consider to be crucial, and which I have formulated with regard to biological entities, must be kept in mind when considering synthetic-biological entities. Because even when cars become obsolescent, and their obsolescence can be remedied through intervention, biological entities more or less remedy their own obsolescence by effecting their own intervention.

If a synthetic-biological entity is, by definition, a biological entity, it will resist obsolescence autonomously, or much more autonomously than a car might (Nicholson, 2013). It would only fail to do so if, focusing on the synthetic part of the entity, we were to introduce some types of control that might prevent this natural dynamic. I cannot, however, conceive of another way of controlling the biological entity that is not based on absolute knowledge of the entity. In other words, intervention into the biological entity or, failing that, manufacturing a biological entity from biological components, i.e., creating a synthetic-biological entity, differs fundamentally from the case of the entity we refer to as a car. Every aspect of the latter is designed, right from the start. By contrast, the biological entity is not designed, and our knowledge of how it functions is in no way complete. This being the case, we find ourselves in uncharted territory, not that I wish to suggest that we are in completely uncharted territory, or that there is no way to control what we are dealing with. There exist various controls of and external interventions into the entity itself, which may render said entity controllable, or perhaps, I should say, increasingly controllable, rather than totally controllable. The synthetic-biological entity is preceded by a biological entity which, as an autonomous system in the process of evolution, requires a certain level of knowledge in order for its self-effected interventions to be controlled (Serrano, 2007).

## Conflict of Interest Statement

The author declares that the research was conducted in the absence of any commercial or financial relationships that could be construed as a potential conflict of interest.

## References

[B1] Danchin A. (2009). Bacteria as computers making computers. FEMS Microbiol. Rev. 33, 3–26. 10.1111/j.1574-6976.2008.00137.x 19016882 PMC2704931

[B2] de Lorenzo V. (2014). From the *selfish gene* to *selfish metabolism*: revisiting the central dogma. Bioessays 36, 226–235. 10.1002/bies.201300153 24419968

[B3] Endy D. (2005). Foundations for engineering biology. Nature 438, 449–453 10.1038/nature04342 16306983

[B4] Moya A. (2014). The Calculus of Life. New York: Springer Verlag (in press).

[B5] Nicholson D. J. (2013). Organisms ≠ machines. Stud. Hist. Philos. Biol. Biomed. Sci. 44, 669–678. 10.1016/j.shpsc.2013.05.014 23810470

[B6] Serrano L. (2007). Synthetic biology: promises and challenges (editorial). Mol. Syst. Biol. 3, 158 10.1038/msb4100202 18091727 PMC2174633

